# The Adipocyte and Adaptive Immunity

**DOI:** 10.3389/fimmu.2020.593058

**Published:** 2020-11-27

**Authors:** Jianfeng Song, Tuo Deng

**Affiliations:** ^1^ National Clinical Research Center for Metabolic Diseases, and Department of Metabolism and Endocrinology, The Second Xiangya Hospital of Central South University, Changsha, China; ^2^ Key Laboratory of Diabetes Immunology, Ministry of Education, and Metabolic Syndrome Research Center, The Second Xiangya Hospital of Central South University, Changsha, China; ^3^ Clinical Immunology Center, The Second Xiangya Hospital of Central South University, Changsha, China

**Keywords:** adipocyte, adaptive immunity, adipokine, T cell, B cell

## Abstract

Not only do Adipocytes have energy storage and endocrine functions, but they also play an immunological role. Adipocytes are involved in adaptive immunity to mediate the pathological processes of a variety of chronic inflammatory diseases and autoimmune syndromes. The adaptive immune response consists of T cell-mediated cellular immunity and B cell-mediated humoral immunity. Obese adipocytes overexpress MHC class II molecules and costimulators to act as antigen-presenting cells (APCs) and promote the activation of CD4^+^ T cells. In addition, various adipokines secreted by adipocytes regulate the proliferation and differentiation of T cells. Adipokines are also involved in B cell generation, development, activation, and antibody production. Therefore, adipocytes play an important role in B cell-mediated adaptive immunity. This review describes how adipocytes participate in adaptive immunity from the perspective of T cells and B cells, and discusses their role in the pathogenesis of various diseases.

## Introduction

Adaptive immunity is characterized by specificity, immunological memory, and self/nonself recognition (1). The function of the adaptive immune system is to recognize, remember and destroy invading pathogens through their antigens, and relieve pathogen-associated toxicities. There are two main mechanisms in the adaptive immune system—humoral immunity and cellular immunity, which are mediated by antibodies and cells respectively. The T and B cells are the major components of adaptive immunity. T cells play a large role in the cellular immune response, while B cells are intimately involved in the humoral immune response.

Adipocytes are the main constituent cells of adipose tissue. Their main function is to store energy in the form of lipid droplets when there is excess energy and to supply energy when the body demands it. In addition to their main functions, adipocytes have endocrine functions and can secrete a variety of adipokines such as leptin, adiponectin, and resistin (2–4). Recently, an increasing number of studies have shown that adipocytes have immunological functions capable of recruiting and activating immune cells. The adipocyte was reported as an antigen-presenting cell (APC) which expresses CD1d and MHC class I and II molecules. Several studies have shown that adipocytes highly express CD1d, which presents lipid antigens to invariant natural killer T (iNKT) cells and stimulates the activation of iNKT cells (5–7). Moreover, like other nucleated cells, adipocytes express MHC class I molecules. However, there is no clear evidence that adipocytes interact directly with CD8^+^ T cells through antigen:MHCI complex. In our recent research, we observed that adipocytes express MHC class II molecules and co-stimulatory molecules CD80/CD86, and that their expression significantly increases in response to high fat diet (HFD) challenges (8). Adipocytes can directly activate CD4^+^ T cells through antigen:MHCII complex in a contact-dependent manner. Simultaneously, adipocytes secrete various cytokines including leptin, resistin, TNF-α and IL-6 to regulate the differentiation and function of T and B lymphocytes.

Adipocytes can regulate adaptive immunity, which is involved with various metabolic diseases. Since there have been many reports on the regulation of metabolic diseases through adaptive immunity (9–11), we focus on how adipocytes regulate adaptive immunity in this review. First, we introduce adipocytes as APCs to participate in T cell-mediated adaptive immune response. Next, we summarize various cytokines produced by adipocytes that regulate the survival, activation and differentiation of B cells. Adaptive immunity mediates the pathological processes of a variety of chronic inflammatory diseases, autoimmune syndromes and cancers. Thus, we discuss the role of adipocytes in adaptive immunity in the context of inflammatory and autoimmune diseases.

## The Role of Adipocytes in T Cell-Mediated Adaptive Immunity

The activation and differentiation of T cells require three signals: antigen presentation, costimulation, and cytokine stimulation. APCs are required for T cell activation. They can process and present antigens to T cells in the form of antigen peptide:MHC molecular complexes, which are recognized by TCR on T cells to provide the first signal for T cell activation. Moreover, APCs highly express co-stimulatory molecules and pair with the corresponding receptor or ligand molecules on the surface of T cells, constituting the second signal for T cell activation. After T cells are fully activated, the further proliferation and differentiation of T cells depends on a variety of cytokines, including IL-2, IL-4, IL-6, IL-10, IL-12, and IFN-γ. In this section, we will describe how adipocytes act as APCs to provide all three signals for T cells activation and differentiation.

### Adipocyte-Mediated Antigen Presentation

Adipocytes express both MHC classes I and II molecules. MHCI molecules are expressed in all nucleated cells and mediate CD8^+^ T cell activation, while MHCII molecules are restricted to antigen-presenting cells (APCs) and induce CD4^+^ T cell activation by antigen presentation. APCs are divided into professional APCs and non-professional APCs. The former includes dendritic cells (DC), monocytes/macrophages, and B lymphocytes, and the latter comprises endothelial cells, epithelial cells and fibroblasts (12). In our previous studies, we found that adipocytes also express MHCII molecules, and that their levels are significantly increased in adipocytes of HFD fed mice (8). In contrast, MHCI-related genes in adipocytes remain unchanged during obesity.

Adipocyte MHCII begins to increase at 2 weeks of HFD, and the expression of pro-inflammatory Th1 marker genes Tbx21 and Ifng in adipose tissue resident T cells (ART) increase at 2–3 weeks following HFD, suggesting that adipocyte MHCII may mediate Th1 cell activation and trigger obesity-induced adipose inflammation. *In vitro* adipocyte-T cell co-culture experiments show that the activation of T cells by adipocytes is dependent on direct contact between adipocytes and T cells and the MHCII expression in adipocytes (8). Large adipocytes (diameter >25 μm) express higher levels of MHCII than small adipocytes (diameter <25 μm) in both ND (normal diet)- and HFD-fed mice. In obesity, large adipocytes are accumulated in adipose tissues and they overexpress MHCII molecules. These hypertrophic adipocytes can function as APCs to activate CD4^+^ ART and instigate adipose tissue inﬂammation, which could cause many obesity-related medical complications (13). Adipocyte-speciﬁc MHCII deficient (aMHCII^−/−^) mice are signiﬁcantly more sensitive to insulin and glucose tolerant than their wild type (WT) littermates when fed with HFD (14). In addition, adipocytes of HFD-fed aMHCII^−/−^ mice exhibit reduced capacity to activate CD4^+^ T cells, as manifested by attenuated secretion of IFN-γ, a major Th1 cytokine (14). Furthermore, adipocyte MHCII has an indirect effect on Tregs in visceral adipose tissue (VAT). aMHCII^−/−^ mice show increased Treg abundance in VAT, compared with WT mice under HFD. *In vitro* experiments show that IFN-γ dose-dependently inhibits Treg differentiation (14). Thus, in the HFD-fed aMHCII^-/-^ mouse model, the drop of IFN-γ may explain the increase of Tregs in VAT. Given that VAT Treg is a negative regulator of adipose inflammation and insulin resistance (15–17), the improved adipose inflammation and insulin resistance in HFD-fed aMHCII^−/−^ mice may result from the increase of Tregs in VAT. Indeed, the preserved insulin sensitivity of HFD-fed aMHCII^−/−^ mice is attenuated by ablation of Tregs in adipose tissue (14). These results indicate that adipocyte MHCII can promote adipose inflammation and insulin resistance. Consistently, adrenomedullin 2 improves adipose insulin resistance by inhibiting the adipocyte MHCII expression in the early stage of obesity (18). HFD-fed adipocyte HIF-1α KO mice show decreased expression of MHCII genes, and can protect themselves from obesity-induced adipose inflammation (19). In summary, the adipocyte can function as APCs to induce CD4^+^ T cell activation and polarization in MHCII and antigen dependent pathway.

Current research on adipocyte MHCII antigen presentation and co-stimulation focuses on obesity and type 2 diabetes (T2D). Therefore, the metabolic diseases we have discussed in this review are obesity and T2D. Since adipocyte-mediated antigen presentation promotes adipose inflammation, which is strongly associated with a variety of metabolic diseases, including nonalcoholic fatty liver disease (NAFLD), atherosclerosis, heart disease, etc., adipocyte-mediated antigen presentation may contribute to these metabolic diseases indirectly.

### Co-Stimulatory Molecule in Adipocyte

TCR recognition of antigen peptide/MHCII provides the primary signal for CD4^+^ T cell activation, while the full activation of CD4^+^ T cells requires the costimulation signal. Costimulatory molecules on the surface of T cells and APCs bind to each other in a receptor–ligand pairing manner. Costimulatory molecules expressed by T cells interacts with its ligands or receptors on the membrane of APCs, resulting in the activation of these cells and thus triggering immune response (20).

Recent studies have reported the role of T cell costimulators in HFD-induced obesity (21), but the contribution of adipocytes in T cell costimulation is still unclear. CD40 (22), CD80 (B7-1), CD86 (B7-2) (8, 23) and HVEM (24, 25) are induced in adipocytes of obese human or mice, and may costimulate adipose resident T cells (ARTs) in obesity. However, studies show that both CD40 knockout mice and CD80/CD86 double knockout mice under HFD feeding exhibit exacerbated adipose tissue inflammation and metabolic disorders. To understand these unexpected results, investigators explored the involvement of other factors that can also influence the phenotype of these mice. After binding with CD40L, CD40 triggers the recruitment of adaptor proteins, the TNFR-associated factors (TRAFs), to activate intracellular signaling (26). The cytoplasmic region of CD40 contains a proximal binding site for TRAF6 and a distal binding site for TRAF2/3/5. Mice that are deficient in CD40-TRAF2/3/5 signaling in MHCII^+^ cells display a similar phenotype as CD40^−/−^ mice under HFD, whereas mice with disrupted CD40-TRAF6 signaling in MHCII^+^ cells are protected against obesity-induced metabolic dysfunction (27). CD40-TRAF2/3/5 and CD40-TRAF6 signaling have opposite effects in obesity-related metabolic disorders. This may explain the unexpected phenotype of CD40^−/−^ mice. In addition, CD80/CD86 double knockout mice have congenital defects in the development of Tregs, which may explain the aggravated adipose inflammation in these mice. Indeed, using antibodies to block both CD80 and CD86 can alleviate adipose inflammation, insulin resistance and fatty liver of diet-induced obese mice (23, 28). Another costimulatory receptor–ligand pair, HVEM-LIGHT, is also involved in the ART activation of DIO mice. LIGHT is expressed in both activated and resting T cells in mice (29). LIGHT binds to HVEM on adipocyte, and promotes the secretion of pro-inflammatory cytokines and chemokines in adipocytes by activating the NF-kB signaling pathway in human and mice (30, 31), thereby inducing the recruitment of T cells and macrophages in adipose tissue. Both HVEM genetical deletion and treatment of HVEM blocking antibodies in HFD-fed mice ameliorates obesity-induced adipose tissue inflammation and metabolic deterioration (24, 32).

These studies have suggested that T cell costimulatory molecules may be involved in the obesity-induced activation of ART and the development of adipose tissue inflammation. However, it is still uncertain whether adipocytes provide the T cell costimulatory signal to activate ARTs during obesity, because no studies have used adipocyte-specific costimulator knockout mice to confirm the function of adipocytes in T cell costimulation. Moreover, several costimulatory molecules have been linked to obesity-induced adipose inflammation and insulin resistance, but it is still unclear which costimulator plays the central role. Further studies are warranted to address these unanswered questions.

### Adipokines That Regulate Activation and Polarization of T Cell

A variety of cytokines secreted by adipocytes can regulate the activation and differentiation of T cells and B cells, and participate in various metabolic and non-metabolic diseases. Since the topic of how adipokines contribute to metabolic diseases has been extensively described in many reviews (33–36), in this review, we focus on non-metabolic diseases.

#### Leptin

Leptin is basically a pro-inflammatory adipokine that directly or indirectly regulates T cells proliferation and differentiation (**Table 1**). As early as 1998, Lord et al. found that leptin promotes the proliferation of naïve and memory T cells and increases the secretion of Th1 cytokines, but suppresses the production of Th2 cytokines (37). Subsequently, it has been reported that leptin constrains the activation and proliferation of Treg cells (38). Mechanism studies have shown that leptin activates the mTOR pathway, thereby exerting a positive effect on CD4^+^ CD25^−^ FOXP3^−^ effector T cells (Teffs), but inhibiting Foxp3 expression and the proliferation of Treg cells (39, 40). Leptin also promotes Th17 responses by inducing the transcription of retinoid-related orphan receptor γt (RORγt), the key transcription factor for Th17 differentiation (41). In addition, leptin has positive effects on the generation, maturation and survival of thymic T cells by reducing their apoptosis (42). Furthermore, leptin increases the secretion of inflammatory cytokines (e.g. IL-6, IL-12 and TNF-α) as well as the expression of chemokine ligands (e.g. CCL3, CCL4 and CCL5) by activating the JAK2–STAT3 pathway in monocytes/macrophages from human or mice (43, 44), thereby indirectly promoting differentiation and adaptive immune response of T cells.

**Table 1:** The effects of adipokines on T lymphocytes.

Adipokines	Naïve CD4^+^ T	Th1	Th2	Th17	Treg	Tfh	CD8^+^ T
Leptin	Proliferation↑	Differentiation↑ Cytokines secretion ↑	Differentiation↓ Cytokines secretion ↓	Differentiation↑ Cytokines secretion ↑	Differentiation↓	Differentiation↑ Cytokines secretion ↑	Activation↑
Adiponectin	Proliferation↓ Apoptosis↑	Differentiation↑↓ Cytokines secretion ↑↓	Differentiation↑ Cytokines secretion ↑	Differentiation↑↓ Cytokines secretion ↑↓	Differentiation↑	Activation↑	Development↑
IL-6	Proliferation↑ Apoptosis↓	Differentiation↓ Cytokines secretion ↓	Differentiation↑ Cytokines secretion ↑	Differentiation↑ Cytokines secretion ↑	Differentiation↓	Differentiation↑	Differentiation↑ Activation↑
TNF-α	Proliferation↑	Differentiation↑ Cytokines secretion ↑ Migration↑	Differentiation↓ Cytokines secretion ↓	Differentiation↑ Cytokines secretion ↑ Migration↑	Differentiation↓	—	Activation↑ Proliferation↑ Migration↑
Resistin	Migration↑	—	—	—	Differentiation↑	—	—
Visfatin	Activation↑	—	—	—	—	—	—

↑ Indicates upregulation, ↓ indicates downregulation, ↑↓ indicates both upregulation and downregulation, － indicates unknown.

Due to its strong effects on T cells, leptin participates in the pathological processes of a variety of inflammatory and autoimmune diseases. In obesity-induced adipose inflammation, leptin stimulates IFN-γ secretion from ART, which leads to an increase in pro-inflammatory Th1 cells and a decrease in anti-inflammatory Tregs in adipose tissue (8). Leptin gene expression in adipocytes is elevated within 1 week of HFD, suggesting that leptin plays a role in initiating the cascade of adipose inflammation. Moreover, because leptin can promote the proliferation of autoreactive T cells and differentiation of pro-inflammatory Th1 and Th17 cells in human and mice, it has been reported to be involved in the induction and progression of IBD (45, 46), multiple sclerosis (47–49), rheumatoid arthritis (50, 51) and systemic lupus erythematosus (41, 52)

#### Adiponectin

Adiponectin has dual effects on T cell function. Several studies have shown that adiponectin is a negative regulator of T cell activity. It has been reported that adiponectin inhibits the proliferation and cytokine production of T cells, and promotes their apoptosis (53). Recent data indicates that adiponectin inhibits Th1 and Th17 differentiation through the upregulation of SIRT1 and PPARγ and inhibition of RORγt (54). It also suppresses IL-17 production from γδ-T cells (55). Therefore, adiponectin ameliorates Th17 cell-mediated autoimmune diseases, including experimental autoimmune encephalomyelitis (EAE) (54) and psoriasiform skin inflammation (55). In a mouse model of abortion, adiponectin increases Treg cell population *via* enhancing Foxp3 expression, thereby improving the pregnancy rate of this model (56). Furthermore, the immunomodulatory effect of adiponectin on T cells is partially mediated by its ability to suppress the allostimulatory capacity of dendritic cells (DCs) (57). Adiponectin suppresses the expression of MHCII and co-stimulators CD80 and CD86, and induces the expression of co-inhibitor PD-L1 in DCs. Adiponectin-treated DCs show a reduced capacity to promote CD4^+^ T cell proliferation and an enhanced capacity to induce Treg expansion in DC-T cell cocultures (57).

However, some studies showed opposite results that adiponectin is a pro-inflammatory adipokine. In human polyclonally activated CD4^+^ T cells, adiponectin treatment results in the increased secretion of IFN-γ and IL-6, phosphorylation of p38 MAPK and STAT4 and expression of T-bet, which indicates a potential function of adiponectin promoting Th1 differentiation (58). Moreover, adiponectin aggravates collagen-induced arthritis (CIA) *via* enhancing Th17 cells and T follicular helper (Tfh) cells response (59). Adiponectin also reduces the apoptosis of lamina propria T lymphocytes (LPL-T) in IBD patients by inducing expression of anti-apoptotic proteins Bcl-xL and Bcl-2, leading to T cell-mediated inflammation (46). Adiponectin also indirectly promotes Th1 and Th17 polarization by activating DCs through PLCγ/JNK/NF-κB signaling pathway (60).

The reason for the discrepancy in effects of adiponectin on T cells is unclear. Adiponectin circulating in plasma has three major forms: trimer, hexamer, and high molecular weight (HMW) multimer (61). Different oligomers activate different intracellular signaling pathways, resulting in significantly different effects (62). It is possible that different oligomers of adiponectin were used in different studies, which results in this discrepancy.

#### IL-6

IL-6 is a pro-inflammatory cytokine that is secreted by various immune cells. Adipocytes also express IL-6. Although adipocytes are not the main source of IL-6 in adipose tissue, IL-6 has been considered as an adipokine (63). IL-6 has different effects on different CD4^+^ T cell subsets. It was reported that IL-6 inhibits Th1 differentiation by upregulating the expression of a suppressor of cytokine signaling,(SOCS)-1, a potent inhibitor of IFN-γ signaling (64). IL-6 also inhibits TGF-β-induced Treg cell’s differentiation (65, 66). However, IL-6 induces the production of IL-4, resulting in increased Th2 polarization (67). In addition, IL-6 is a crucial cytokine for lineage commitment to Th17 cells. IL-6 promotes Th17 differentiation by activating STAT3, which upregulates the expression of RORγt and RORα (68, 69). Furthermore, IL-6 is a positive regulator of Tfh cells (70), which is supported by the observation that the early differentiation of Tfh cells is severely impaired in IL-6 deficient mice (71).

Although the regulatory effect of IL-6 on CD4^+^ T cells has been extensively studied, the effect of IL-6 on CD8^+^ T cells is still poorly understood. IL-6 may positively regulate CD8^+^ T cell function. IL-6 was found to promote the generation of CD8^+^ cytotoxic T cells (72). It was reported that IL-6 induces the differentiation of naïve CD8^+^ T cells into IL-21-producing CD8^+^ T cells, which improve IgG isotype switching in B cells during influenza virus infection (73). This is a new function for IL-6 in the prevention of viral infection. Furthermore, IL-6 promotes the differentiation of IL-22-producing CD8^+^ T cells, a CD8^+^ T cell subset with antitumor function (74).

#### Other Adipokines

In addition to the adipokines mentioned above, some other secreting factors of adipocytes, including resistin, visfatin, and TNF-α, also regulate T cell function. Resistin induces activation of Src and PI3K in human CD4^+^ T lymphocytes and serves as a chemokine for these cells (75). Moreover, resistin indirectly enhances Treg expansion through the regulation of DCs, in which interferon regulatory factor (IRF)-1 pathway is suppressed by resistin (76). Visfatin is an adipokine that upregulates the activation of T cells. It promotes the production of IL-1β, IL-1Ra, IL-6, IL-10, and TNF-α and the expression of costimulatory molecules CD80, CD40 and ICAM-1 (CD54) in monocytes, thereby stimulating the activation of T cells (77). It is worthy to note that although visfatin is expressed in adipose tissue, its expression is higher in bone marrow, the liver and muscles (78). Additionally, in the adipose tissue, visfatin is not only expressed in adipocytes. Studies have found that visfatin is mainly produced and released by macrophages in white adipose tissues (79). Therefore, adipocytes may not be the major source of visfatin expression. TNF-α is an important immunomodulatory cytokine, which plays a critical role in regulating the proliferation, differentiation, and apoptosis of T cells, the generation of memory T cells, and maintenance of immune tolerance (80). It has been reported that TNF-α is secreted by adipocytes and other immune cells (81, 82). However, whether adipocytes produce TNF-α is still controversial.

## The Role of Adipocytes in B Cell-Mediated Adaptive Immunity

Similar to T cell activation and differentiation, B cell activation and differentiation also requires three signals. But unlike T cells that recognize antigens presented by APCs, B cells recognize free antigens through B cell receptor (BCR). B cells specifically recognize antigens through BCR, generating the first signal for B cell activation. B cells are per se professional APCs. B cells internalize the antigen bound by BCR and process the antigen to form an antigen peptide–MHCII complex, which is presented to antigen-specific Th cells. After Th cells are activated, they express high levels of co-stimulatory molecules and combine with matched ligands or receptors on the surface of B cells, which provides the second signal for B cell activation. Activated B cells express multiple cytokine receptors, and proliferate and differentiate into antibody-forming cells under the action of cytokines that are secreted by activated T cells or other cells. For B cell activation, the role that adipocyte plays on the two key signals, antigen recognition and costimulation has not yet been reported, but some studies have reported that several adipokines play a role in the development and differentiation of B cells. Here we discuss the role that adipocytes play in regulating B cell-mediated adaptive immune responses through secreted cytokines (**Table 2**).

**Table 2:** The effects of adipokines on B lymphocytes.

Adipokines	Pro-B	Pre-B	Immature B	Mature B	Plasma B
Leptin	Development↑	Development↑	Development↑	Development↓ Cytokines secretion↑	Antibody production↑
Adiponectin	Development↓	Development↓	—	Cytokines secretion↑	—
IL-6	—	—	—	Differentiation↑ Proliferation↑	Antibody production↑
Visfatin	—	Colony formation↑	—	Activation↑ Migration↑	—
BAFF	—	—	—	Survival↑ Maturation↑ Proliferation↑	Antibody production↑
Other soluble factors	Development↓	Development↓	—	—	—

↑ Indicates upregulation, ↓ indicates downregulation, — indicates unknown.

### Leptin

In addition to regulating T lymphocytes mediated immune responses, leptin plays an important role in the regulation of B cell development and function. Deficiency of leptin signaling in ob/ob and db/db mice leads to the decrease of B cells in bone marrow and peripheral blood, while intraperitoneal injection of leptin in ob/ob mice restores the number of bone marrow B cells (83), suggesting that leptin plays a critical role in supporting B cell development. Fasted mice, characterized by low serum leptin levels, show decreased pro-B and immature B cells and increased mature B cells in bone marrow (84). Leptin receptor is expressed on B cells, suggesting a direct effect of leptin on B cells (85). However, the fasting-induced atrophy of bone marrow B cells is reversed by intracerebroventricular leptin injection, indicating that leptin may indirectly regulate B cell development through the central nervous system (86). In addition to its effects on the regulation of B cell development, leptin suppresses apoptosis and induces cell cycle entry of B cells by upregulating the expression of Bcl-2 and Cyclin D1 (87). Moreover, leptin stimulates human B cells to secrete proinflammatory (TNF-α and IL-6) and anti-inflammatory (IL-10) cytokines, *via* activation of JAK2/STAT3 and p38MAPK/ERK1/2 signaling pathway (88). Interestingly, leptin-induced production of TNF-α, IL-6 and IL-10 in B cells from aged individuals are significantly higher than that in B cells from young individuals (89). Furthermore, leptin promotes immunosenescence of human B cells. Leptin treatment results in declined immunoglobulin class switch and influenza vaccine-specific IgG production in human B cells (90).

### Adiponectin

Adiponectin has two receptors, ADIPOR1 and ADIPOR2. Both are abundantly expressed on the surface of circulating B cells (91). However, the immunomodulatory effects of adiponectin on B lymphocytes are not very clear. It has been reported that adiponectin inhibits B lymphopoiesis in long-term bone marrow cultures. This effect is highly dependent on the presence of both stromal cells and early B lineage precursors in the cultures (92). Adiponectin deficient mice treated with dextran sulfate sodium (DSS) present more significant B cells infiltration in colons and appear more severe colitis than WT littermates, indicating that adiponectin may suppress B cell-mediated inflammatory response in DSS-induced colitis (93). Moreover, adiponectin stimulates B cells to secret a peptide, PEPITEM, which specifically inhibits the migration of CD4^+^ and CD8^+^ memory T cells (94). Further studies are guaranteed to address the detailed role of adiponectin in regulating B lymphocytes function.

### Other Adipokines

Leptin and adiponectin are exclusively expressed in adipocyte. Some other adipokines that are secreted by both adipocytes and other types of cells also have regulatory effects on B cells. These adipokines include visfatin, B cell activation factor (BAFF), and IL-6. Visfatin was previously called ‘pre-B cell colony-enhancing factor (PBEF)’, since it enhances pre-B-cell colony formation in the presence of both IL-7 and SCF (78). Visfatin is a potent chemotactic factor for B cells and promotes B cell migration *in vitro* cell culture (77). BAFF, also known as ‘B lymphocyte stimulator (BlyS)’, promotes B cell proliferation, survival, maturation and immunoglobulin secretion (95, 96). The production of BAFF is upregulated in obese human adipocyte, and it may activate B cells in adipose tissue during obesity (97). IL-6 was originally named ‘B-cell stimulatory factor 2 (BSF-2)’. This name reflects its function to induce differentiation of activated B cells into antibody (Ab)-producing cells (98). IL-6 is abundantly secreted by adipocytes during obesity, and aggravates obesity-induced insulin resistance (99). In addition, some unidentified soluble factors secreted by adipocytes inhibit B lymphopoiesis (100, 101). These factors may mediate the decline of B lymphopoiesis in aged and obese individuals, and both conditions are characterized by increased fat accumulation in bone marrow.

## Conclusion and Future Directions

**Figure 1:**
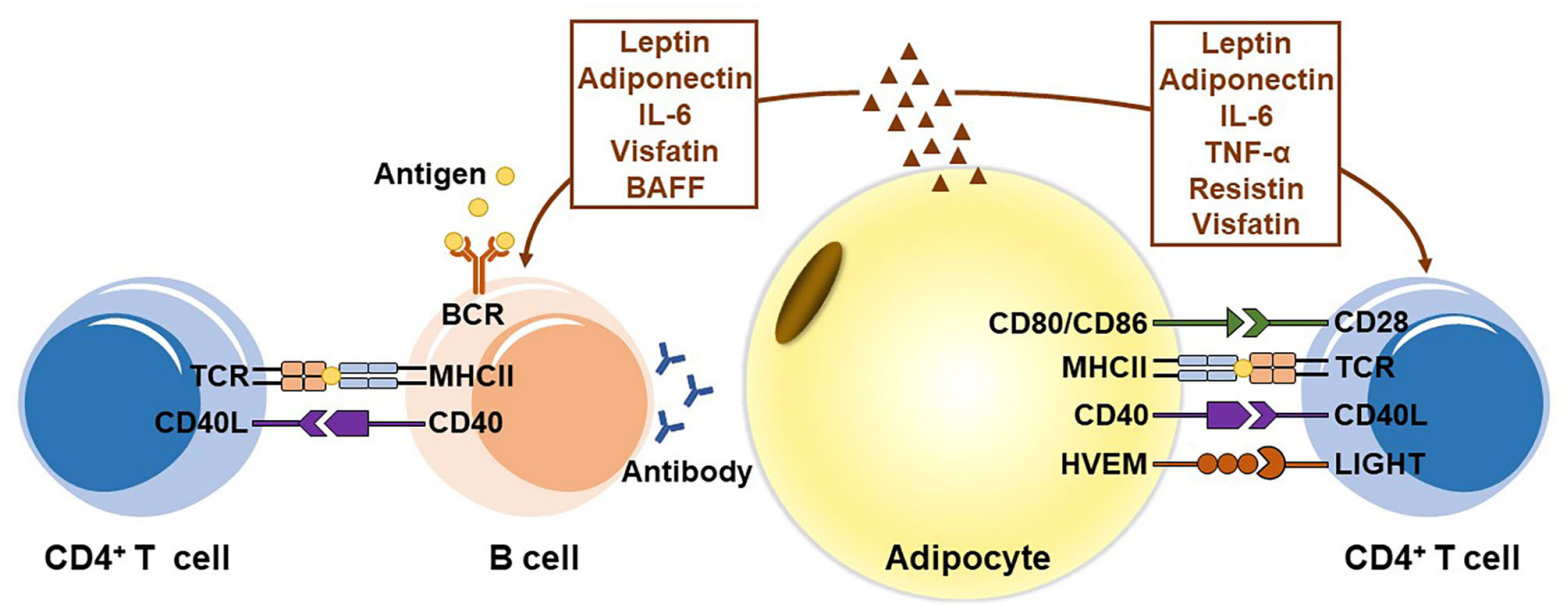
The role of adipocytes in adaptive immunity. Adipocytes express MHC class II molecules and several T cell costimulators to act as antigen-presenting cells (APCs), and induce the activation of CD4^+^ T cells in visceral adipose tissue during obesity. In addition, adipocytes secrete various adipokines, including leptin, adiponectin, IL-6, TNF-α, resistin, and visfatin, to regulate the proliferation and differentiation of T cells. In B cell-mediated humoral immunity, adipocytes modulate B cell generation, development, aging, activation and antibody production mainly by secreting adipokines, including leptin, adiponectin, IL-6, visfatin, and BAFF.

Recently, the immunological function of adipocytes has received increasing attention. Mounting evidence indicates that adipocytes play an important role in adaptive immunity (**Figure 1**). Adipocytes can serve as APCs to regulate T cell-mediated adaptive immunity. The MHCII molecules are expressed in adipocytes and their expressions are upregulated during obesity, providing the first signal for CD4^+^ T cell activation. Simultaneously, adipocytes of obese mice and humans overexpress several costimulatory molecules, including CD40, CD80 (B7-1), CD86 (B7-2) and HVEM. Those constimulators are associated with obesity-induced adipose inflammation and metabolic disorders. However, studies exhibited conflicting results and did not provide convincing data from adipocyte-specific knockout mouse models. Therefore, it is too early to draw a conclusion that adipocytes provide the key costimulatory signal for ART activation. In addition, adipocytes secrete various cytokines, such as leptin, adiponectin, IL-6, resistin, visfatin and TNF-α, which regulate the proliferation and differentiation of T cells and are involved in many chronic inflammatory and autoimmune diseases. In B cell-mediated humoral immunity, adipocytes regulate B cell development, proliferation, differentiation, activation and antibody production through secreted adipokines.

In the past few years, although great progress has been made in understanding the mechanism and function of adipocytes in adaptive immunity, there are still many imperative questions remaining to be answered in this emerging field. Many studies have implied the existence of specific antigens to activate T cells in adipose tissue, but up until now, no any adipose antigen has been reported. In addition, although many T cell costimulators have been linked to obesity-induced adipose inflammation and insulin resistance, the key co-stimulator(s) in obesity-induced ART activation are not known. Identifying of antigen(s) which are recognized by ART in obesity and the key co-stimulatory signaling in ART activation may provide new targets for specifically block obesity-induced adipose inflammation. We found that obesity induces MHCII expression in adipocytes and causes adipocytes to become APCs. But it is still unclear whether all adipocytes or just a subset of adipocytes are converted to APC in obesity. If the latter is true, further studies are warranted to investigate the origin and features of this special adipocyte subpopulation. Finally, compared with the number of studies which concern adipocytes regulating the function of T cells, there are far fewer studies on adipocytes regulating the function of B cells. Except for adipokines, we know little about how adipocytes regulate the B cell-mediated adaptive immune response. Future studies on the mechanisms by which adipocytes regulate B cell function will help us better understand the physiological and pathological functions of adipocytes in B cell-mediated humoral immunity.

## Author Contributions

JS and TD wrote and revised the manuscript. All authors contributed to the article and approved the submitted version.

## Funding

This work was supported by grants from the National Natural Science Foundation of China (81770868), the Major Research plan of the National Natural Science Foundation of China (91742103), and the Project of Innovation-Driven Plan of Central South University (2020CX015).

## Conflict of Interest

The authors declare that the research was conducted in the absence of any commercial or financial relationships that could be construed as a potential conflict of interest.
