# Corrigendum: The Adipocyte and Adaptive Immunity

**DOI:** 10.3389/fimmu.2021.784125

**Published:** 2021-10-08

**Authors:** Jianfeng Song, Tuo Deng

**Affiliations:** ^1^National Clinical Research Center for Metabolic Diseases, and Department of Metabolism and Endocrinology, The Second Xiangya Hospital of Central South University, Changsha, China; ^2^Key Laboratory of Diabetes Immunology, Ministry of Education, and Metabolic Syndrome Research Center, The Second Xiangya Hospital of Central South University, Changsha, China; ^3^Clinical Immunology Center, The Second Xiangya Hospital of Central South University, Changsha, China

**Keywords:** adipocyte, adaptive immunity, adipokine, T cell, B cell

In the published article, there was an error in **Affiliation 1**.

Instead of “National Clinical Research Center for Metabolic Diseases, Key Laboratory of Diabetes Immunology (Central South University), Ministry of Education, Metabolic Syndrome Research Center, and Department of Metabolism and Endocrinology, The Second Xiangya Hospital of Central South University, Changsha, China”, it should be split into two separate affiliations “National Clinical Research Center for Metabolic Diseases, and Department of Metabolism and Endocrinology, The Second Xiangya Hospital of Central South University, Changsha, China” and “Key Laboratory of Diabetes Immunology, Ministry of Education, and Metabolic Syndrome Research Center, The Second Xiangya Hospital of Central South University, Changsha, China”.

The authors apologize for this error and state that this does not change the scientific conclusions of the article in any way. The original article has been updated.

## Publisher’s Note

All claims expressed in this article are solely those of the authors and do not necessarily represent those of their affiliated organizations, or those of the publisher, the editors and the reviewers. Any product that may be evaluated in this article, or claim that may be made by its manufacturer, is not guaranteed or endorsed by the publisher.

